# Novel preparation of functional β-SiC fiber based In_2_O_3_ nanocomposite and controlling of influence factors for the chemical gas sensing

**DOI:** 10.1038/s41598-022-11000-6

**Published:** 2022-05-04

**Authors:** Zambaga Otgonbayar, Young Jun Joo, Kwang Youn Cho, Sang Yul Park, Kwang Youl Park, Won-Chun Oh

**Affiliations:** 1 Department of Advanced Materials Science and Engineering, Hanseo University, Seosan-si, Chungnam, 356-706 Korea; 2 College of Materials Science and Engineering, Anhui University of Science and Technology, Huainan, 232001 People’s Republic of China; 3 Korea Institutes of Ceramic Engineering and Technology, Gyeongsangnam-do, Soho-ro, Jinju-Si, South Korea; 4 Daeho I&T, Gyeongsangnam-do, Changwon-si, 51338 Korea

**Keywords:** Environmental sciences, Materials science

## Abstract

The gas sensing ability of a pure β-SiC fiber is limited due to its low-sensitivity and selectivity with poor recovery time during a gas sensing test. The combination of functional β-SiC fibers with metal-oxide (MO) can lead to excellent electronic conductivity, boosted chemical activity, and high reaction activity with the target gas and β-SiC–In_2_O_3_ sensor material. Influence factors such as amounts of MO, current collectors, and gas species (CO_2_, O_2_ and without gas) for the gas sensing ability of β-SiC–In_2_O_3_ nanocomposite were determined at standard room temperature (25 °C) and high temperature (350 °C) conditions. The gas sensing ability of the functional β-SiC fiber was significantly enhanced by the loading of In_2_O_3_ metal-oxide. In addition, the MO junction on the β-SiC fiber was mainly subjected to the Si–C–O–In bond sensor layer with an effective electron-transfer ability. The gas sensing mechanism was based on the transfer of charges, in which the sensing material acted as an absorber or a donor of charges. The sensor material could use different current- collectors to support the electron transfer and gas sensing ability of the material. A 1:0.5M SiC–In_2_O_3_ coated Ni-foil current collector sensor showed better sensing ability for CO_2_ and O_2_ gases than other gas sensors at room temperature and high temperature conditions. The sensing result of the electrode was obtained with different current density values without or with gas purging conditions because CO_2_ and O_2_ gases had electron acceptor properties. During the gas sensing test, the sensor material donated electrons to target gases. The current value on the CV graph then significantly changed. Our obtained sample analysis data and the gas sensing test adequately demonstrated that MO junctions on functional β-SiC fibers could improve the sensitivity of a sensor material and particularly upgrade the sensor material for gas sensing.

## Introduction

Gas detection tests are used in important tests for engine capacity and chemical plants, especially hazardous gases^1–5^. Sensing and detecting gases or molecules on a wide bandgap nanomaterial mainly depends on the physical and chemical properties of the material A wide bandgap semiconductor material is more useable and stable under high voltage and temperature. Silicon carbide (β-SiC) has excellent thermal stability and strong electrical conductivity under high temperature conditions (25–900 °C)^6–10^. Conventional pure silicon and carbon materials cannot resist extreme conditions. Thus, they are unacceptable for hazardous gas sensing performance. The strong resistance of electrochemical performance and stability properties of β-SiC fiber have changed the usability levels of silicon and carbon materials. However, β-SiC-based sensor materials also have disadvantages such as low-sensitivity and selectivity with poor recovery time during sensing^11–14^. Such disadvantages of the β-SiC-based sensor materials mentioned above are mainly due to the thickness of the sensor which has a thin-film and a miniature contact area. Recently, 2D-structured materials have been widely used for gas sensing experiments due to their large surface area to volume ratios, large absorbing capacities for gas molecules, and strong surface activities. Two-dimensional SiC materials have good gas sensing properties at high temperatures (above 300 °C)^15^. However, the sensing activity of pure β-SiC fiber is not ideal due to the low reaction activity between the gas and the surface of the electrode. The most commonly used method to increase SiC activity is to combine β-SiC fiber and MO (metal oxide) to form a gas-sensing material. This method can bring excellent electronic conductivity and boost chemical activity (enhanced chemical activity, interactivity of the gas and electrode surface).

Metal oxide (MO)-based sensor materials have a chemiresistive sensor feature because they are cheap and easy to operate, and are especially strongly related to instrument analysis^16,17^. In our research, we combined β-SiC fibers with In_2_O_3_, which has a wide bandgap energy (3.6–4.0 eV), good optical and electrical properties, and strong stability and usability features in gas sensing applications. The sensitivity of MO and β-SiC fiber is mainly affected by the morphological structure and shapes of nanomaterials^18,19^. The gas sensing mechanism is based on the transfer of charges, with the sensing material acting as absorbers or donors of charges. The charge transfer between the gas molecule and the sensing material will cause changes in sensing material properties. The gas sensor works by bridging two electrodes (source and drain) with sensing materials and passing current through them. Gas detection can be realized by monitoring current changes upon exposure to the target gas environment under a constant voltage. For a conductance type gas sensor, both high sensitivity and a fast recovery rate are desirable^20^. The gas sensing performance of the electrode is influenced by the gas flow rate, humidity, temperature, and sensing material type and dimension factors. There are several approaches to improve the performance of a sensor such as using programming temperature, using UV light in the sensor, and using nanoparticles as catalysts to improve absorption and selectivity^21^.

In this study, we synthesized In_2_O_3_ nanoparticles via a low temperature, hydrothermal method using indium nitrate. The β-SiC–In_2_O_3_ binary nanocomposite was synthesized following the same procedure. As-prepared nanocomposites were calcined at 400 °C to obtain perfect morphological structures. The development of a gas sensing material with very sensitive and very selective sensing is important to construct a wearable gas sensing device. The characteristics of the nanocomposite were evaluated to define its surface state, ratio, and pore distribution. The as-prepared nanomaterials were then subjected to gas sensing tests. Gas selectivity was determined using different target gaseous products.

## Experiment part

### Preparation of In_2_O_3_

Pure In_2_O_3_ was prepared by hydrothermal method. First, 50 ml 0.3M Na_2_CO_3_ and In(NO_3_)_3_ solutions were prepared separately. These two solutions were then mixed and stirred (400 rpm) for 3 h at 50 °C. To improve the supersaturation of In^3+^ ions in the solution, ultrasonication was conducted for 1 h at frequency of 50 kHz. The solution was transferred into a 100 ml Teflon lined autoclave and kept at 190 °C for 12 h. Precipitated powder was collected by centrifugation at 8,000 rpm for 40 min. The collected powder was washed with DI-water and ethanol to remove impurities and dried at 80 °C in an electric oven. The powder was ground with an agate mortar and calcined in a sealed crucible at 400 °C for 2 h with a heating speed of 10 °C/min.

### Preparation of β-SiC–In_2_O_3_

Binary nanocomposites were prepared using a hydrothermal method following an ultrasonication process. In detail, 1 M β-SiC fiber solution was prepared using a mixture of ethanol and DI-water (25 ml: 25 ml) and stirred for 1.5 h at room temperature. Meanwhile, an In_2_O_3_ solution was prepared using the same solvent. The as-prepared solutions were mixed and stirred at 50 °C for 3 h to prepare a homogeneous solution followed by ultrasonication for 1 h at a frequency of 50 kHz. The ultrasonication process resulted in rapid nucleation of β-SiC–In_2_O_3_ and improved solute transfer. The solution was transferred to a Teflon lined autoclave and kept at 190 °C for 12 h. Precipitated powder was then collected by centrifugation at 8000 rpm for 40 min. The collected powder was washed with ethanol to remove impurities and dried at 80 °C for 10 h in an electric oven. The powder was ground with an agate mortar and calcined in a sealed crucible at 400 °C for 2 h with a heating speed of 10 °C/min to obtain well-structured SiC–In_2_O_3_ binary nanocomposites. The molar ratio of In_2_O_3_ in the binary nanocomposite varied from 0.5M to 0.1M (x = 0.5M, 0.3M, 0.1M). It was possible to study how the percentage of In_2_O_3_ could affect the electrochemical ability and morphological state of the final sample.

### Characterization

#### Sample characterization

The crystal structure of the nanocomposite was analyzed by XRD (SHIMADZU XRD-6000) equipped with a Cu Ka X-ray source (1.5406 Å). The resistivity of each binary nanocomposite was analyzed with EIS and a three-electrode system. The surface morphology and element percentages were analyzed with SEM (JSM-5600 JEOL, Akishima, Tokyo, Japan) incorporated with EDX. The vibrational mode of the sample was tested with a confocal-Raman imaging system using a 532.13 nm excitation laser (Renishaw in Via Reflex, NRS-5100). The size and shape of the nanomaterial were analyzed with TEM (Hitachi H9500, Tokyo, Japan). Elements in the material, surface structure, and electronic structure were tested with XPS (PHI 5000 Versa Probe).

#### Gas sensing test

The sensor response of the binary nanocomposite was tested using different gas purging systems. The sensor device was prepared with the following steps. First, as-prepared SiC- xIn_2_O_3_ was mixed with binding material (ethyl cellulose) and used to make a fine slurry with an ethanol dispersant. The amount of the ethyl cellulose is equal to 10% of the weight of the used samples. The as-prepared slurry was spread on the current collector and the blade was used to adjust the thin-film on the collector surface. The coated electrode was dried at 30 °C in an oven to make a uniform gel-layer on the current collector. The as-prepared electrode was placed in the gas-sensing reactor. The temperature in the reactor was room temperature (25 °C) and high temperature (350 °C). The gas-flow rate was controlled with a mass-flow controller at 6 kg/cm^2^ for 120 min. During the gas flow condition without gas, the CV graph was measured by using a PGP201 potentiostat (A41A009). The current density in the CV graph was obtained from the ratio between the density amount before purging the gas and that after purging the gas.

## Results and discussion

### XRD pattern and EIS test

**Figure 1:**
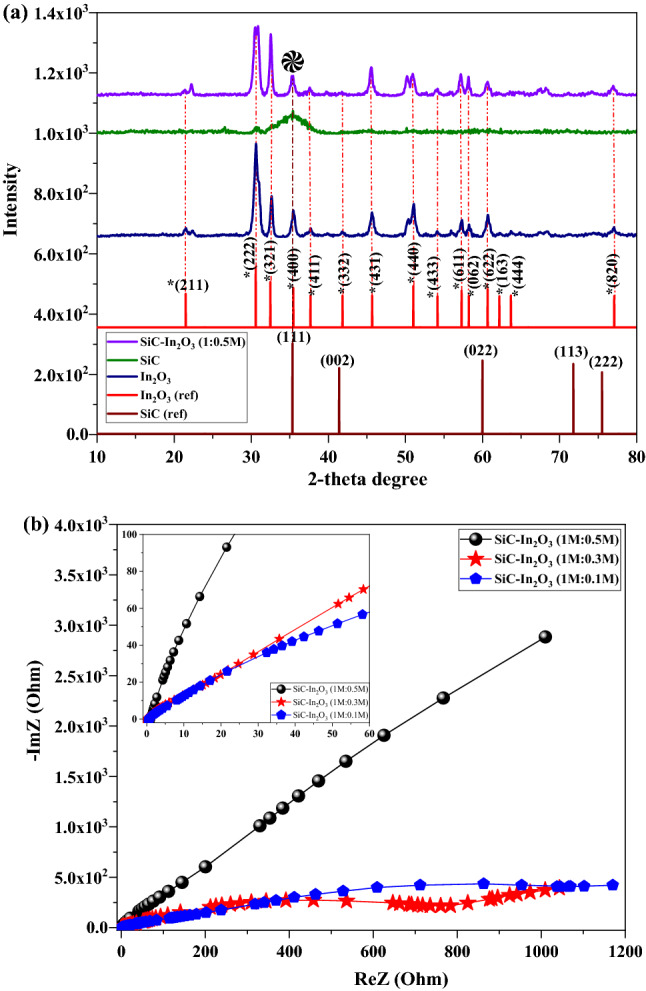
(**a**) XRD pattern; and (**b**) EIS test of the In_2_O_3_, β-SiC fiber and SiC-In_2_O_3_ nanocomposite.

The crystal structures and XRD patterns of the nanocomposites are shown in Fig. 1a. The reference data of SiC and In_2_O_3_ was obtained from High Score Plus software. The black diffraction peak indicated the XRD pattern of In_2_O_3_, which well-matched with the JCPDS No. 0.6-1416 card. The sharp peak and FWHM decrease indicated that the as-prepared In_2_O_3_ nanocomposite had a decent crystal structure. The broad diffraction peak at 35.51° 2-theta degrees indicated the (111) crystal plane of the β-SiC fiber (JCPDS No. 29-1129). In the XRD pattern of SiC–In_2_O_3_, the peak of the In_2_O_3_ was significantly changed to low-sharpness, indicating that MO could cover the surface of β-SiC-fibers with successful interactions between each nanocomposite. In SiC–In_2_O_3_, the XRD peak SiC and In_2_O_3_ overlapped at 35.51° 2-theta degree and this peak strongly related to the SiC-fiber. The grain size of the nanocomposite was calculated by following the Debye-Scherer equation

$$D=\frac{(K\lambda )}{(\beta\, cos \theta )}$$

where d is the particle size, λ is the wavelength of X-ray radiation (1.5406 Å), K is a crystallite size factor (constant factor K = 0.9), β is the full-width at half-maximum (FWHM) of the peak (in radians) and 2*θ* is the Bragg angle). The grain size of the pure In_2_O_3_ was 3.34 nm, β-SiC fiber was 1.23 nm, and the β-SiC–In_2_O_3_ had 1.97 nm grain size.

Figure 1b shows the electrochemical resistance spectra of β-SiC–In_2_O_3_ binary nanocomposites containing different amounts of In_2_O_3_. The resistance and conductivity of the samples are mainly related to their semicircle profile in the EIS test. From the above results, it was found that the 0.1M In_2_O_3_ loaded nanocomposite had a small semicircle with low resistance properties. It is effective for the electrochemical coefficient of the β-SiC–In_2_O_3_ nanocomposite during the test. The β-SiC-fiber had good electrochemical conductivity. The effectiveness of the In_2_O_3_ to β-SiC was lacking, and In_2_O_3_ did not reduce or change the fundamental properties of the β-SiC fiber. The EIS profiles of the samples were remarkably changed when the loading amount of In_2_O_3_ was increased from 0.1M to 0.5M, which might be due to the interconnection between the β-SiC fiber and MO.

### EDX and SEM analysis

**Figure 2:**
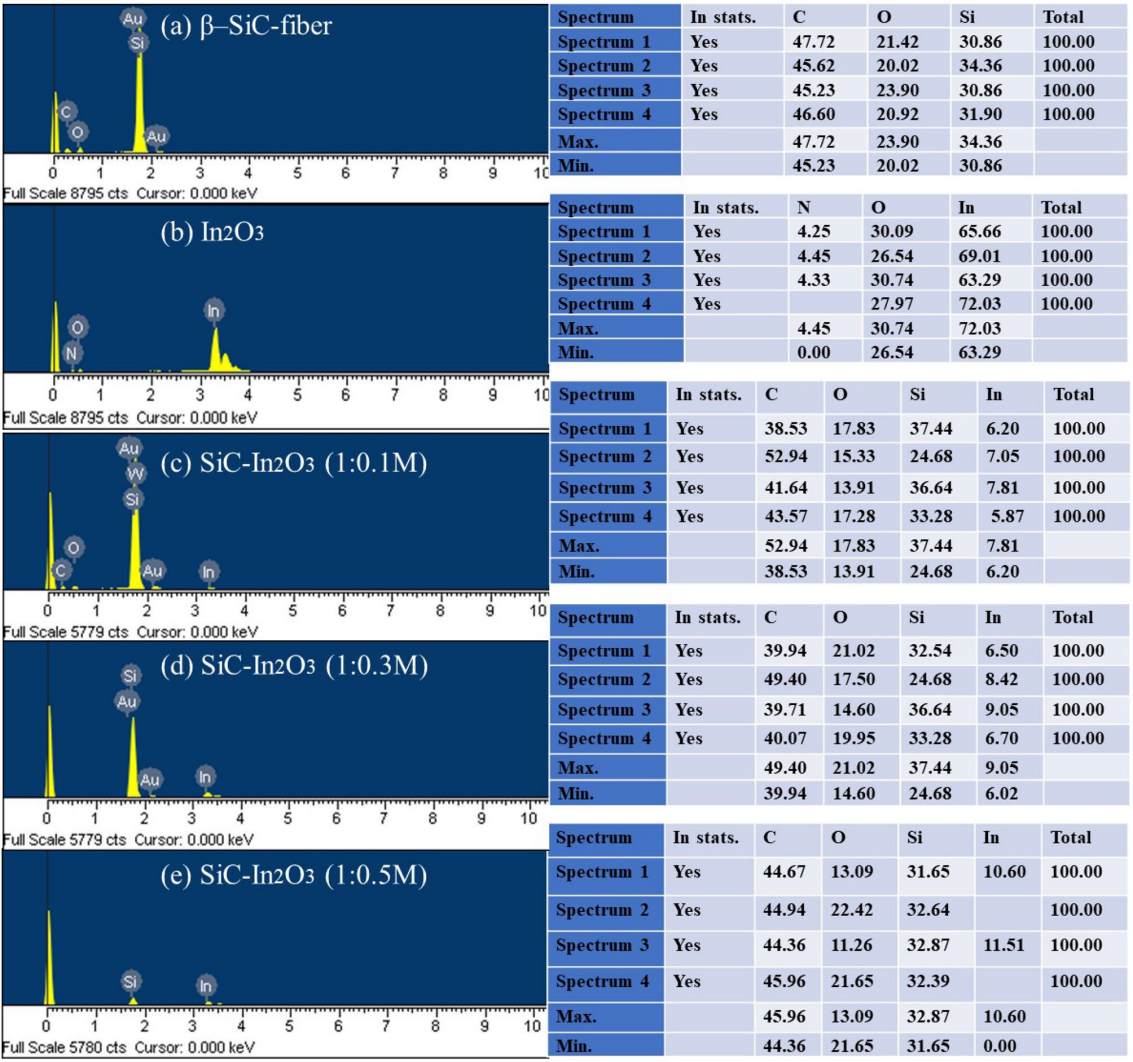
EDX analysis of the In_2_O_3_, β-SiC fiber and SiC-In_2_O_3_ nanocomposite.

The surface state, morphology, and atomic amount of each element in the nanocomposite were analyzed by EDX and SEM. From the EDX results, the main elements were found in high amounts as shown in Fig. 2. The N-element was found in the EDX result of In_2_O_3_. This might be derived from the precursor material used for In_2_O_3_ synthesis. After combining In_2_O_3_ with β-SiC fiber followed by calcination, there was no amount of N-element in the result. Other elements were not found either, confirming that the final sample was successfully synthesized without impurities using a simple hydrothermal method.

**Figure 3:**
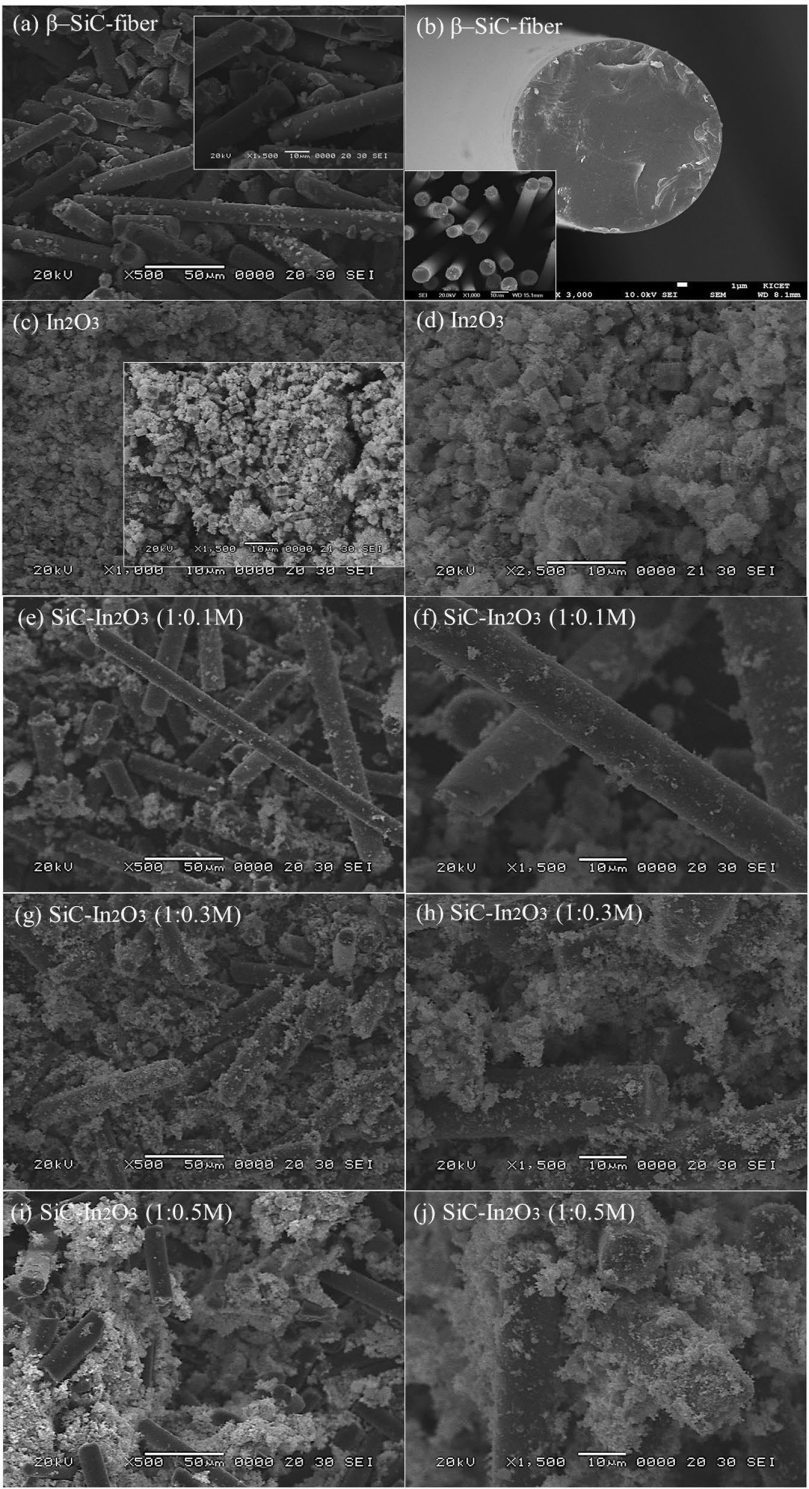
SEM analysis of the (**a**,**b**) β-SiC fiber, (**c**,**d**) In_2_O_3_ and (**e**–**j**) SiC-In_2_O_3_ nanocomposite.

The surface morphology and profile of the as-prepared nanomaterials were analyzed by SEM. Pure β-SiC fibers had a smooth and clear surface with all nanoparticles regularly agglomerated as shown in Fig. 3a,b. The thickness of the nanoparticles was the same, with a width of 11.3 nm and non-similar length. The smooth surface of the β-SiC fiber supports the location of In_2_O_3_ on the surface, which can prevent MO from irregularly spreading on the surface. Pure In_2_O_3_ had irregularly shaped primary nanoparticles such as cubes and spheres, with all primary nanoparticles were agglomerated. The size of the cube-shaped nanoparticles was approximately 3.23 μm. The surface was smooth and thicker than sphere-shaped (in Fig. 3c,d). The surface images of the different amount of metal oxide loaded β-SiC–In_2_O_3_ nanocomposite were displayed in Fig. 3e–j. The metal oxide agglomeration on the β-SiC fibers were non-similar due to the loading amounts. Especially, the agglomeration of the metal oxide was becoming strong in 1:0.5M β-SiC–In_2_O_3_ (in Fig. 3i,j). Figure 3f shows the SEM image of the 0.1M In_2_O_3_ loaded β-SiC fiber nanocomposite. The loading amount of the metal oxide was less than amount of the pure β-SiC fiber, and irregular shaped metal oxide cannot homogeneously spread and covered the SiC-fiber surface, which is confirmed by SEM result. And the thickness pure β-SiC fiber was 15–16 μm in Fig. 3b, the thickness of the SiC–In_2_O_3_ (1:0.5 M) was 18–20 μm (as shown in Fig. 3j). The thickness of the SiC–In_2_O_3_ (1:0.3M) was 17–18 μm, and the SiC–In_2_O_3_ (1:0.1M) had almost same thickness as a pure β-SiC fiber. In SEM images of β-SiC–In_2_O_3_, sphere-shaped In_2_O_3_ was mainly observed on smoother β-SiC fibers. All nanoparticles were agglomerated, resulted in a porous structure profile with different sizes. Moreover, pure In_2_O_3_ well-dispersed onto the surface, which can effectively increase the gas sensing performance.

### Molecular dimensional analyses by Raman spectra and TEM

**Figure 4:**
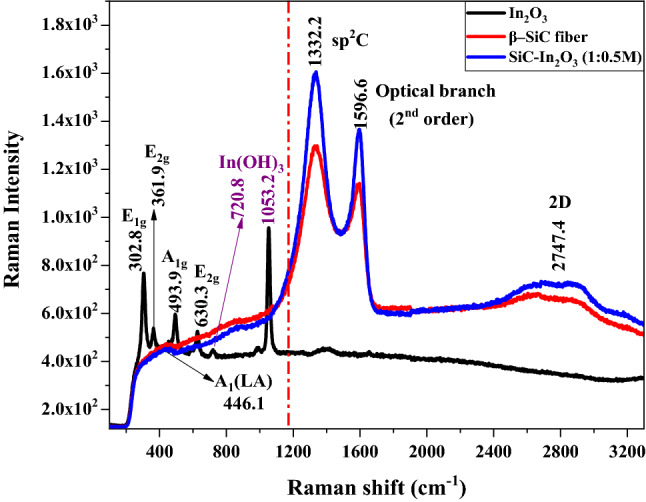
Raman spectra of In_2_O_3_, pure β-SiC fiber and β-SiC-In_2_O_3_ nanocomposite (thermal annealing at 400 °C).

The symmetric motion, chemical bonding, and interaction of nanomaterials were analyzed by Raman spectroscopy. Vibrational frequencies are specific to the symmetric motion of molecules and chemical bonds in the final nanocomposite^22,23^. Full Raman spectra are displayed in Fig. 4. Raman peaks of pure MO were observed below 1200 cm^−1^ Raman shift regions. A total of six Raman peaks appeared in the In_2_O_3_ nanocomposite. The first four peaks were related to E_1g_, E_2g_, and A_1g_ Raman active-modes. Indium hydroxide Raman peaks appeared in the 720.8 cm^−1^ and 1053.2 cm^−1^ frequency regions. These peaks might be due to the conversion of small amounts of In ions to In(OH)_3_ during the synthesis process^24^. Pure β-SiC had two-sharp Raman peaks at 1332.2 cm^−1^ and 1596.6 cm^−1^ regions related to *sp*^2^-hybridized carbon and the optical branch of the second-order Raman spectra, respectively. One-broad peak was obtained at 2747.4 cm^−1^ Raman shift region. It was classified to 2D symmetric mode which can be obtained from the overtone motion of TO-phonons due to activation by double resonance scattering. After combining the β-SiC-fiber with MO, no MO-peak appeared in Raman result, although the peak intensity was increased. Such increases in the Raman intensity are related to a particular mode of vibration that appears in a specific bond to allow a specific Raman active mode. In otherwise, it is related to the expression of supressing and dominating bonds which are formed at the specific frequency energy.

**Figure 5:**
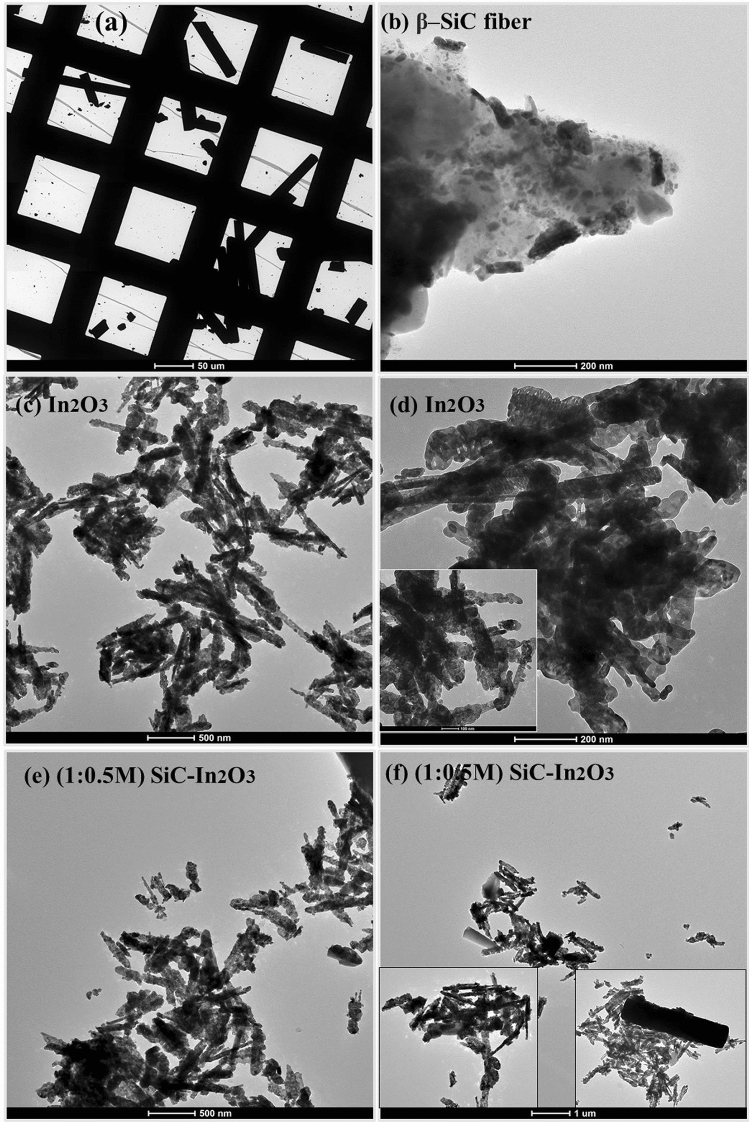
TEM images of the (**a**,**b**) pure β-SiC fiber, (**c**,**d**) In_2_O_3_ and (**e**,**f**) β-SiC-In_2_O_3_ (1:0.5 M) nanocomposite (thermal annealing at 400 °C).

The size and shape of the nanomaterial were analyzed by TEM. Obtained images are shown in Fig. 5. In Fig. 5a,b rod-shaped with short length and long-length β-SiC-fibers were obtained; the long-length SiC-fibers were dominant, which is more favourable for MO and allows uniform distribution on the surface. TEM images of In_2_O_3_ revealed that particles were agglomerated and stacked and created the spherical and grain-shaped particles. The agglomerated particles had cleavage steps, indicating a nonsmoothed surface (Fig. 5c,d). In β-SiC–In_2_O_3_, the most MO wrapped the β-SiC-fiber surface and there was less agglomeration on the surface. MO nanoparticles were obtained as light black-grey coloured images. β-SiC fibers were obtained as dark black colored rod-shaped images and the widths of the fibers were identical. The lower agglomeration of MO on β-SiC fibers can have favourable electron-transfer action during an electrochemical test. To summarize, the simple hydrothermal method could be used to synthesize well-spread In_2_O_3_ on the surface of β-SiC fibers. This provides favourable conditions for efficient electronic conduction and good electrochemical operation.

**Figure 6:**
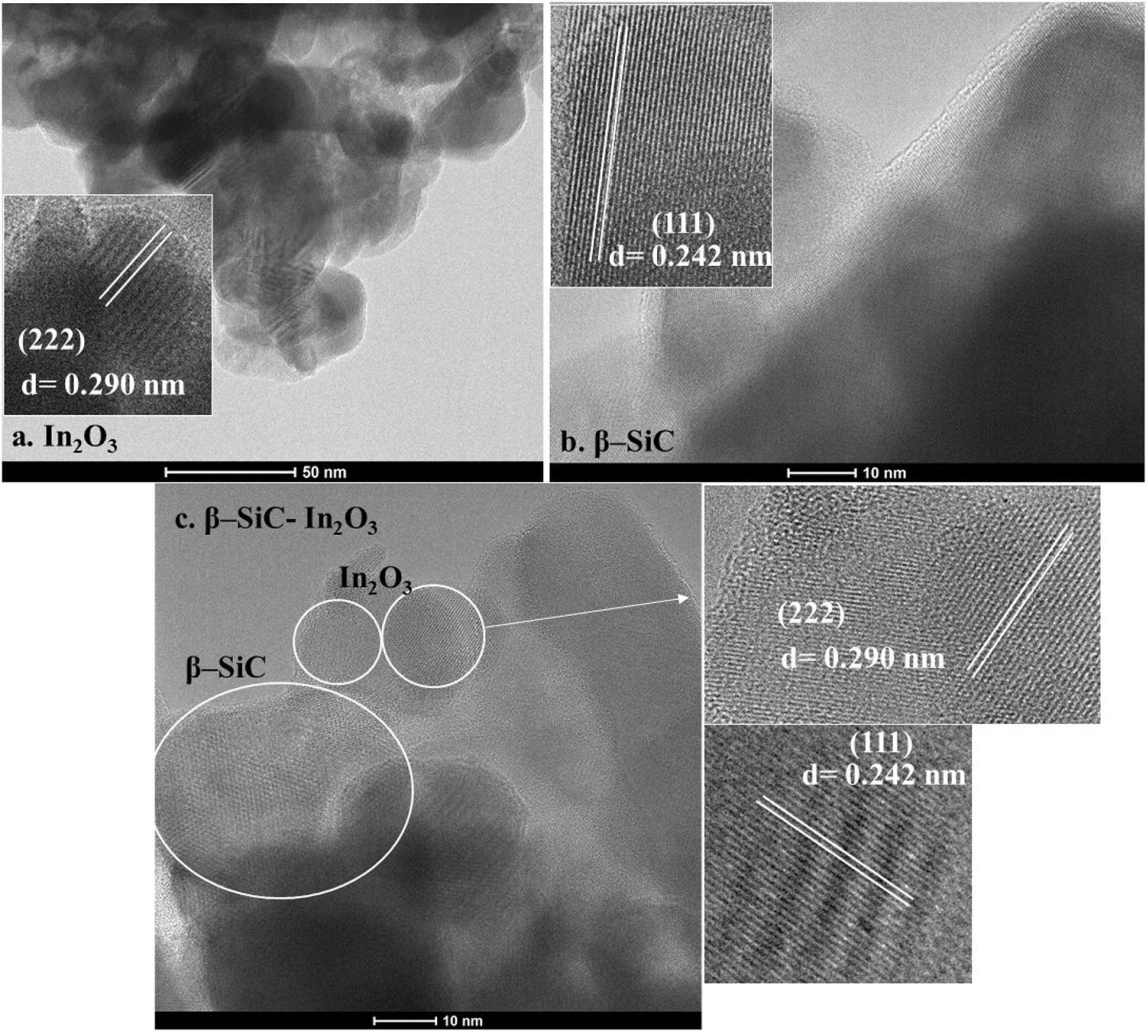
HRTEM images of the (**a**) β-SiC fiber, (**b)** In_2_O_3_ and (**c**) β-SiC-In_2_O_3_ nanocomposite.

**Figure 7:**
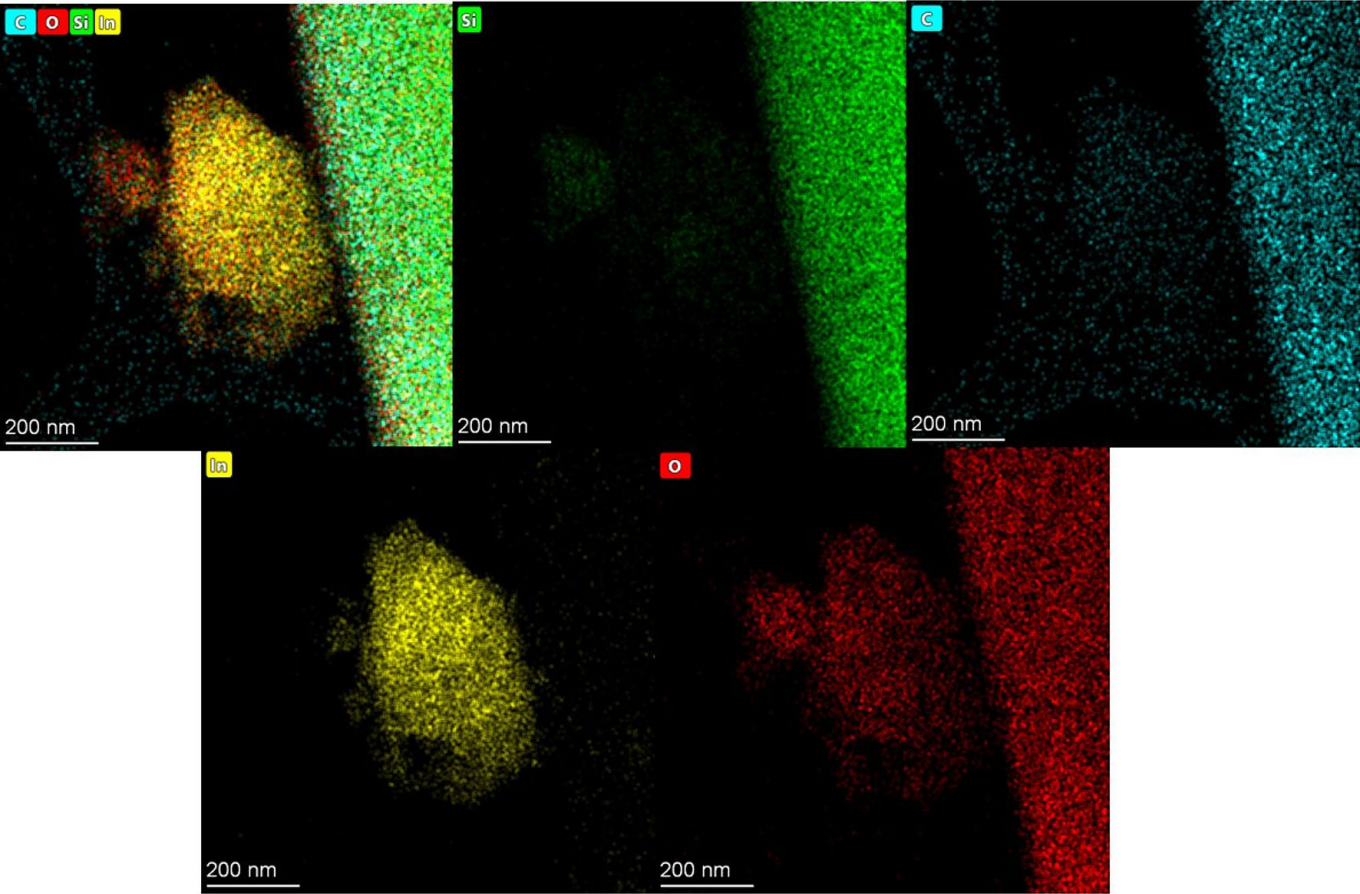
EDS mapping of the SiC-In_2_O_3_ nanocomposite.

The interplane structure of the β-SiC, In_2_O_3_ and β-SiC–In_2_O_3_ nanocomposite were evaluated by the HRTEM analysis, and the obtained images were displayed in Fig. 6. In HRTEM, the d-spacing value and the (hkl) indexes of each nanocomposite. The lattice fringes were perpendicular each other in HRTEM images. The d-spacing values were 0.290 nm and 0.242 nm on pure β-SiC and In_2_O_3._ Those all d-spacing values were corresponding to the (222) and (111) crystal facets, as shown in Fig. 6a–c. Moreover, energy-dispersive X-ray spectroscopy (EDS) result also confirms the presence of each elements in the nanocomposite. The EDS element mapping analysis further indicated the co-existence of β-SiC and In_2_O_3_ and all images were well-displayed in Fig. 7. EDS elemental mappings of β-SiC–In_2_O_3_ revealed the coexistence of Si, C, In and O in the nanocomposite. The corresponding elemental mapping spatial distribution for nanocomposite and the uniform dispersion of these elements further confirmed the successful construction of β-SiC–In_2_O_3_ nanocomposite.

### Chemical bonding and XPS analysis

**Figure 8:**
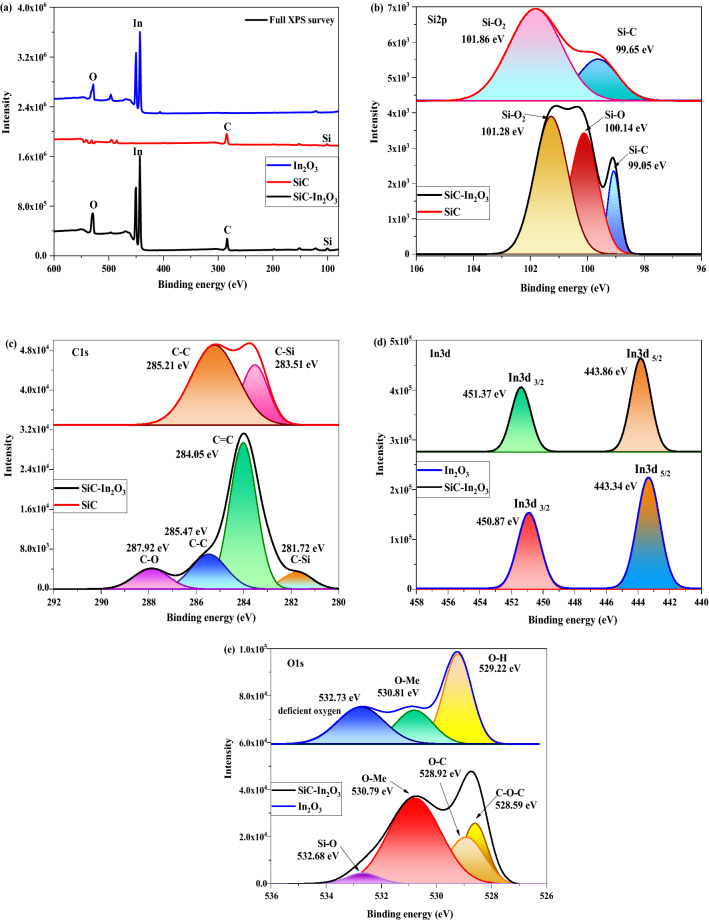
Full XPS surveys of the β-SiC, In_2_O_3_ and β-SiC-In_2_O_3_ nanocomposite (**a**), Si (**b**), C (**c**), In (**d**) and O (**e**).

The element-composition and surface state of the nanomaterials were examined by the XPS. Full XPS survey spectra (Fig. 8a) showed the coetaneousness of Si, C, In, and O in β-SiC fiber, In_2_O_3_ and β-SiC–In_2_O_3_. The Si2p XPS spectra indicate that three electronic structures and chemical bonding states, Si–O, Si–O_2_, and Si–C are on the surface of the β-SiC–In_2_O_3_ nanocomposite (Fig. 8b). The XPS of Si2p on β-SiC fiber further analyzed to evaluate the interfacial bond of the nanocomposites. In β-SiC, two peaks were obtained which assigned to the bonding state of the Si–C and Si–O_2_. The binding energy states were shifted due to the introduction of In_2_O_3_, which is affecting the local environment. The binding energy of Si–C bonding is located at 99.05 eV regions. The binding energies of Si-O_2_ and Si–O obtained in the 101.28 and 100.14 eV regions, respectively, had higher intensities than the Si–C XPS peak of β-SiC–In_2_O_3_. The appearance of Si–O was higher than Si–C bonds, indicating that Si–O form of Si existed on the surface of the β-SiC fiber. Additionally, the O-element appearance can be derived from In_2_O_3_ which is located on the surface of the β-SiC-fiber ^25^. Figure 8c shows the C1s XPS spectrum of the β-SiC-fiber and β-SiC–In_2_O_3_ as-well-as the possible deconvolution spectra of the C-element. As is shown, two peaks appeared in XPS of β-SiC which related to the C–C, C–Si bonding. On the contrary, totally four different bonding appearances were obtained in β-SiC–In_2_O_3_ spectra: C–O, C–C, C=C, and C–Si in the surface. The C–C and C=C bonding derived from the SiC and the peak intensities of C–C and C=C bonding were higher than that of C–O bonding. The C–Si bonding energy was obtained at 281.72 eV region, indicating the metal appearance of the β-SiC-fiber ^25^. The peak intensity of C–O was quite similar to that of C–Si. Both oxygen and silica were derived from MO, showing successful interaction between MO and SiC-fibers. The XPS spectrum of In3d (in Fig. 8d) was deconvoluted into two peaks on pure In_2_O_3_ and β-SiC–In_2_O_3_^26^. In3d XPS peak of In_2_O_3_ was broader than In3d XPS peak of In(OH)_3_, which is confirmed by the reference data. In addition, the location of the peak slightly shifted due to the interfacial connection of the β-SiC and In_2_O_3,_ moreover, the charge density around the In atoms increased. Finally, O1s XPS was analyzed on pure In_2_O_3_ and β—SiC–In_2_O_3_ nanocomposites, and the integrated graph is shown in Fig. 8e. Compared with the pure In_2_O_3_, four different chemical bonding states were detected ^26^. In the spectrum, Si–O, O–Me, C–O–C, and O–C bonding at 532.68, 530.79, 528.59 and 528.92 eV binding energy regions were observed.

The main characteristic peaks were obtained that can expressed the successful junction of the nanocomposites. In detail, Si–O and O–Me bonds are attributed to the contribution of MO. O–Me bond peak describe the metal-oxide (O–In bond of In_2_O_3_), and Si–O bond describe that SiC-fiber had chemical reaction with In_2_O_3_ metal-oxide. O–C bond indicated that C-element of SiC and O-element of In_2_O_3_ was chemically bonded. In addition, Si–O bond obtained in Si2p XPS graph. Those all obtained XPS peaks described that β-SiC fiber and In_2_O_3_ had successful connection each other.

In addition, the peak shift phenomenon observed in each XPS analysis that expressed the charge density, electron configuration and environment condition of the elements were changed after the junction between β-SiC fibers and In_2_O_3_ MO, and the change of the chemical bonds. XPS results confirmed that each element had interconnection and that In_2_O_3_ metal oxides successfully junctioned with β-SiC fibers and chemical bonds on the surface.

### Gas sensing performances

**Figure 9:**
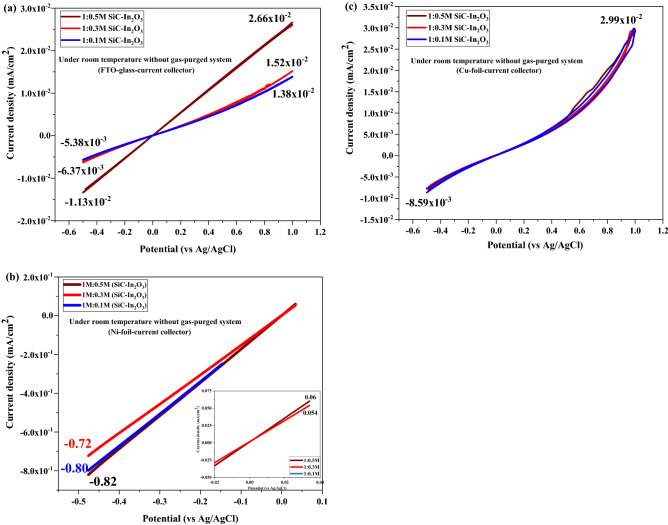
CV test of the as-prepared electrode in room temperature (25 °C) without gas-purging conditions on (**a**) FTO glass, (**b**) Ni foil, and (**c**) Cu foil current collector.

The gas sensing performance of xIn_2_O_3_ loaded with β-SiC-fibers was tested without gas and with (O_2_ or CO_2_) gas purging. The CV profile was examined using a PGP201 potentiostat (A41A009). The test was recorded under a (− 0.5 V) to (+ 1 V) potential range with a (1 A) to (− 1 A) current. Cyclic voltammetry is one of the most commonly used electrochemical analysis techniques. The gas sensor material was used with three-different current collectors: Cu foil, FTO glass, and Ni foil. Figure 9a,b,c shows the results of the CV test of as-prepared electrodes without gas purging. Among them, the Ni-foil current collector drastically supported the electron transfer and electrochemical performance of the SiC–In_2_O_3_ nanocomposite coated sensing material. In Fig. 9a, the high current density was 2.66 × 10^–2^ mA/cm^2^ on 1:0.5 M SiC–In_2_O_3_ coated FTO sensor. The current density value was significantly reduced on 1:0.3 M SiC–In_2_O_3_ and 1:0.1 M SiC–In_2_O_3_ nanocomposite was coated with sensor material. The current value of pure β-SiC fibers was approximately 3 × 10^–6^ mA/cm^2^, which might be due to the fact that pure β-SiC fiber material had low reaction activity and low electrochemical performance at room temperature. The results of CV tests confirmed that the combination of MO and β-SiC-fibers drastically improved the electron transfer and electrochemical performance of the final nanocomposite material. In Fig. 9b, the CV graph of the Ni-foil coated sensor shows a high current density value. The Ni foil current collector has more favourable compatibility with the SiC–In_2_O_3_ nanocomposite. It drastically supported the electrochemical performance of the sensor material. A higher current value of 6 × 10^–2^ mA/cm^2^ was found for the 1:0.5 M SiC–In_2_O_3_ sensor material (in Fig. 9b). The current value changeability between each electrode was not high, indicating that Ni-foil current collector had more stable properties on our synthesized β-SiC–In_2_O_3_ binary nanocomposite. The current density value of sensor material coated Cu foil was quite higher than of coated FTO glass as shown in Fig. 9c. However, the CV graph had a zig-zag profile. This indicated that the sensor material was not properly coated on the Cu-foil surface, leading to an irregular interconnection. These CV results indicated that sensor materials based on different electrical collectors coated with 1:0.5 M SiC–In_2_O_3_ had good electroconductivity and good electrochemical ability, suggesting that these materials might have high gas sensitivity.

**Figure 10:**
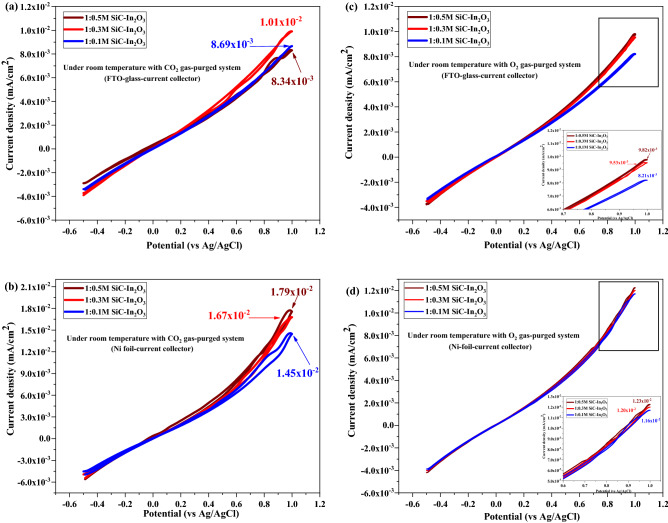
CV test of the as-prepared electrode under CO_2_ and O_2_ gas-purging conditions at normal room temperature (25 °C).

Figure 10 shows the results of the cyclic voltammetry test of the as-prepared gas sensor material under CO_2_ and O_2_ purging conditions at room temperature (25 °C). The gas sensing test on In_2_O_3_ and SiC-fiber under room temperature is common suggested test method (27–28). The gas sensor material was coated on FTO glass and Ni foil current collectors. Figure 10a and b display the gas sensing performance of the coated FTO glass and the Ni foil current collector under CO_2_ gas purging conditions. Current density values of 1:0.5 M β-SiC–In_2_O_3_ were 8.34 × 10^–3^ mA/cm^2^ and 1.79 × 10^–2^ mA/cm^2^, respectively. The current change variation of the gas sensor was different due to conductivity. In the case of Ni foil, it had a porous structure to support the location/coating ability of the material, thus contributing to the electron transport potential. The sensing ability of the sensor material under O_2_-gas is displayed in Fig. 10c and d. Current density values of 1:0.5 M β-SiC–In_2_O_3_ were 9.82 × 10^–3^ mA/cm^2^ and 1.23 × 10–^2^ mA/cm^2^, respectively. Under O_2_ gas purging conditions, the variation of current density was higher than for CO_2_ gas detection. This indicated that the sensor material might have more excellent and effective sensing ability for O_2_ gas sensing at standard room temperature (25 °C) when the sensor material uses a Ni-foil current collector.

The gas sensing test was mainly realized by current change upon exposure to the target gas environment under a constant voltage^29,30^. Under room temperature conditions, activation of the electrode was less. Nevertheless, all sensor materials showed good sensing for CO_2_ and O_2_ gases. The current change variation on 1:0.5 M β-SiC–In_2_O_3_ was higher than those of the other two gas sensor materials at standard room temperature. This provided evidence that this material had a more active sensing ability and that the reaction of gas on the electrode surface was more dynamic. The current change variation on 1:0.1M β-SiC–In_2_O_3_ was lower than other high amount of MO loaded β-SiC-binary nanocomposites, indicating that 0.1M In_2_O_3_ irregularly spread on the surface of the β-SiC fiber and could not activate the sensing ability due to the lack of electron exchange.

**Figure 11:**
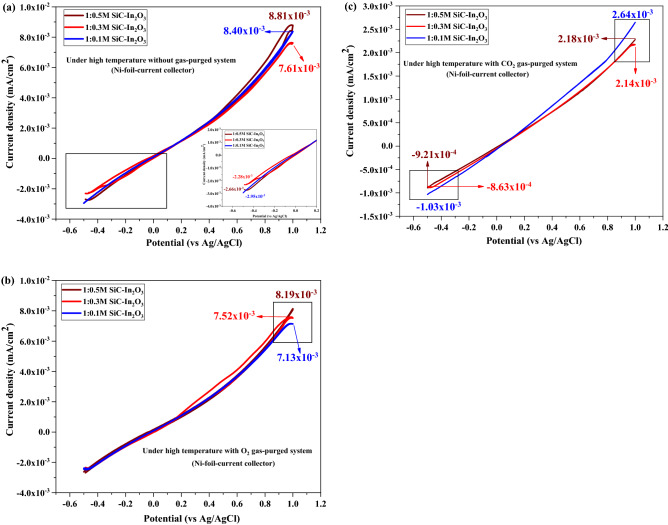
CV test of the as-prepared electrode under high temperature (350 °C) heat treatment with (**a**) without gas purging, (**b**) O_2_ gas-purging and (**c**) CO_2_ gas purging system.

**Figure 12:**
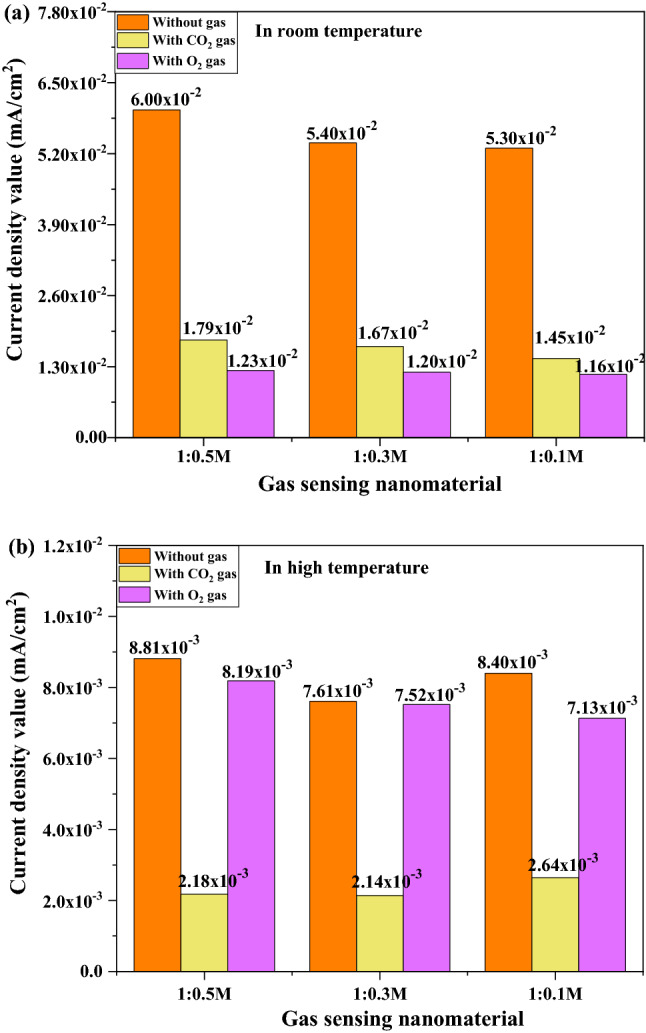
Current density value of the gas sensor with gas and without gas under (**a**) room temperature (25 °C) and (**b**) high temperature conditions (350 °C).

**Figure 13:**
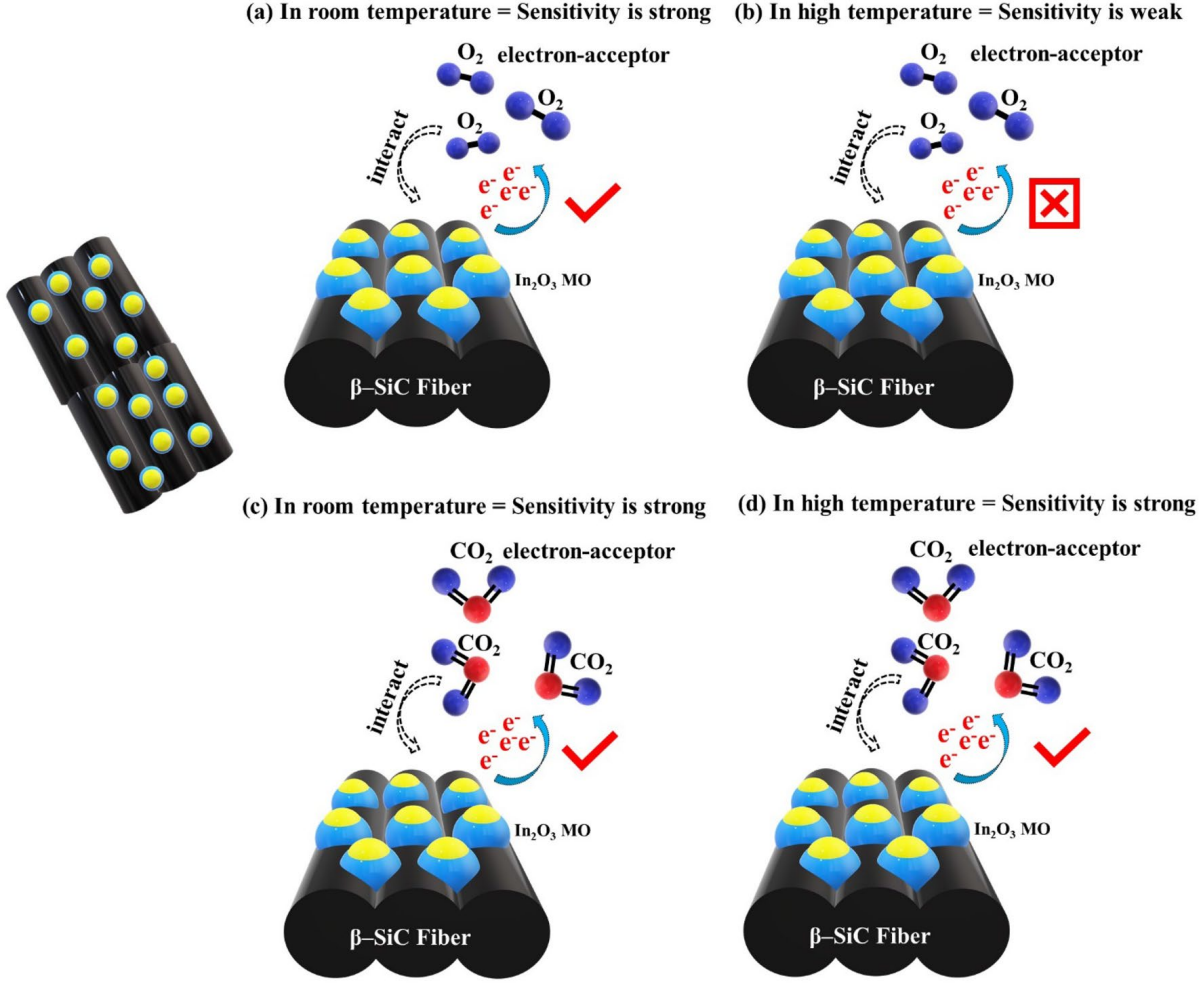
Schematic illustration of the CO_2_ and O_2_ gas sensing performance on the β-SiC-In_2_O_3_ sensor at (**a**,**c**) room temperature (25 °C) and (**b**,**d**) high temperature (350 °C).

The SiC-based gas sensor material had strong activation under high-temperature. In addition, the combination of β-SiC fibers with MO can bring excellent electronic conductivity and boost chemical activity (enhanced chemical activity and interactivity of the gas and electrode surface)^31,32^. Figure 11 shows the results of the electrochemical test of the coated Ni-foil current collector under high temperatures without or with a gas purged state. Under high temperatures, differences among current density values of three different electrodes were not high, suggesting that these prepared electrodes could show quite similar sensing ability. However, the 1:0.5 M SiC–In_2_O_3_ nanocomposite coated electrode had a high current density value (Fig. 11a). Figures 11b and c show electrochemical responses of CO_2_ and O_2_-gas with x-amount of In_2_O_3_ loaded β-SiC fiber electrodes. The 1:0.5 M β-SiC–In_2_O_3_ electrode had high conductivity for O_2_ gas but low-conductivity for CO_2_ gas. Figure 12a and b displays the highest current density value of each gas sensor material without or with a gas purging condition at room temperature (25 °C) and high temperature (350 °C). The unit of current density value is mA/cm^2^. The sensor material had quite strong sensitivity for O_2_ at room temperature as displayed in a bit graph. O_2_ gas had an electron acceptor behaviour. Oxygen gas strongly interacted with the surface of the sensor material. At a high temperature, a strong sensing ability of SiC–In_2_O_3_ sensor was observed for CO_2_ gas. The possible gas sensing reactions on β-SiC–In_2_O_3_ nanomaterial at room and high temperatures are displayed in Fig. 13.

The gas sensing mechanism is based on the transfer of charges, in which the sensing material acts as an absorber or donor of charges. Charge transfer between the gas molecule and the sensing material will cause changes in sensing material properties. Gases such as O_2_ and CO_2_ tend to receive electrons from the sensor surface. The oxidizing gas (receiver) can increase the resistance of the sensor surface and reduce the resistance of the sensor by reducing the gas (donor). Gases such as O_2_ tend to receive electrons from the surface of the sensor, which is an oxygen-dominated gas that takes electrons from the surface of the metal oxide and converts them into ions that can be rapidly absorbed on the surface of a metal-oxide sensor (Fig. 13a and b). As a result, electrons on the surface become trapped, which increases the height of the potential barrier. On the other hand, it affects the surface conductivity of metal oxides or electron conduction. In the case of CO_2_, it has a linear bond and a stable structure with no lone pair of electrons (Fig. 13c and d). The CO_2_ gas react with surface electrons of the gas sensors and made a form of (CO_2_^-^). During a sensing test, surface electrons of the β-SiC–In_2_O_3_ sensor are used to sense CO_2_, or CO_2_ gas receives electrons from the gas sensor so that the current density value (mA/cm^2^) of the gas sensor is significantly lower than that of a normal system or a no gas-purged system.

To conclude, the electron transfer ability between the target gases and sensor material strongly defines the sensing ability of β-SiC–In_2_O_3_. Furthermore, the active parts of the surface affect the reaction between the gas and the surface on the sensor surface. β-SiC–In_2_O_3_ nanocomposites contain varying amounts of In_2_O_3_ metal-oxide, which makes it possible to determine how they affect activities of gaseous materials. The high load of metal oxides strongly supports the electrochemical performance of β-SiC fibers, resulting in the formation of a high electron density Si–C–O–In bond sensor layer. The charge transfer process then becomes more active under the influence of the interface structure. In addition, functional β-SiC fibers had an abundant surface area on which MO can be homogeneously distributed. All factors mentioned above adequately explained the surface modification of the β-SiC fiber, the change of electron-transfer activity, and the gas sensing ability.

## Conclusion

A β-SiC–In_2_O_3_ nanocomposite containing new and unique properties was synthesized by the ultrasonication-method along with the hydrothermal method. The ultrasonication process was one of the leading techniques to achieve rapid nucleation of β-SiC–In_2_O_3_ and improve solute transfer. The morphological state, molecule interaction, and crystal-structure of the nanocomposite were analyzed by XRD, SEM, TEM, EDX, Raman spectroscopy, XPS, and EIS. The gas sensing ability of the β-SiC–In_2_O_3_ nanocomposite was determined in terms of influencing factors such as the amounts of metal oxide, current collectors, and gas species (CO_2_, O_2_, and without gas) at standard room temperature (25 °C) and high temperature (350 °C) conditions. The gas sensing ability of the SiC fiber was significantly enhanced by the loading of In_2_O_3_ metal-oxide. In addition, the metal-oxide junction between SiC fibers was mainly due to the Si–C–O–In bond sensor layer with an effective electron-transfer ability. The electron transfer ability between the target gases and sensor material strongly defines the sensing ability of β-SiC–In_2_O_3_. Furthermore, active parts of the surface can affect the reaction between the gas and the surface on the sensor surface. Our obtained data such as nanocomposite characteristics and gas sensing ability for CO_2_ and O_2_ gases adequately confirmed the successful junction of In_2_O_3_ onto the β-SiC fiber. In conclusion, our proposed sample preparation method and selected gas sensing material junctions drastically upgraded the sensing performance of the β-SiC fiber and the sensor material.
